# Association of NLRP3 rs35829419 and rs10754558 Polymorphisms With Risks of Autoimmune Diseases: A Systematic Review and Meta-Analysis

**DOI:** 10.3389/fgene.2021.690860

**Published:** 2021-07-22

**Authors:** Zubo Wu, Suyuan Wu, Tao Liang

**Affiliations:** ^1^Department of Pediatrics, Union Hospital, Tongji Medical College, Huazhong University of Science and Technology, Wuhan, China; ^2^Department of Clinical Laboratory, Union Hospital, Tongji Medical College, Huazhong University of Science and Technology, Wuhan, China

**Keywords:** NLRP3, single nucleotide polymorphisms, autoimmune diseases, susceptibility, meta-analysis

## Abstract

The existing knowledge about the association between NLRP3 rs35829419/rs10754558 polymorphisms and susceptibility to autoimmune diseases (AIDs) remains controversial. Herein, a meta-analysis was performed to evaluate such association. We searched databases for relevant studies published in English up to February 2021. Stata14 was used to assess the odds ratio (OR). As for NLRP3 rs35829419, no significant association to overall AIDs was found in three genetic models [A vs. C: OR (95%CI) = 0.89 (0.69–1.14); AC vs. CC: 1.00 (0.77–1.30); AA/AC vs. CC: 0.93 (0.71–1.20)]. However, subgroup analysis by disease type showed that NLRP3 rs35829419 A allele may have a significant protective effect on rheumatoid arthritis (RA) susceptibility [A vs. C: 0.74 (0.57–0.96)]. NLRP3 rs10754558 polymorphism contributes to significantly reduce the risk of AIDs in the allelic model [G vs. C: 0.78 (0.71–0.87)], homozygote co-dominant model [GG vs. CC: 0.63 (0.51–0.77)], heterozygote co-dominant model [GC vs. CC: 0.78 (0.66–0.91)], dominant model [GG/GC vs. CC: 0.73 (0.63–0.84)], and recessive model [GG vs. GC/CC: 0.73 (0.62–0.88)]. In the subgroup analysis by ethnicity, association was observed between the NLRP3 rs10754558 G allele and AIDs in Latin Americans, but not in European, Arabian, or Asian populations. Stratification by disease type showed a significant association of the NLRP3 rs10754558 G allele with type 1 diabetes (T1D), RA, and systemic lupus erythematosus (SLE), but not with celiac disease (CD), multiple sclerosis (MS), or myasthenia gravis (MG). This meta-analysis suggests that the NLRP3 rs10754558, but not rs35829419, polymorphism is associated with susceptibility to AIDs, especially in Latin American individuals.

## Introduction

Autoimmune diseases (AIDs) are characterized by a host immune response against self-antigens that damages host tissues. Autoimmune diseases are caused by a combination of genetic factors and environmental factors (Luppi et al., 1995). In particular, the role of genetic factors has been confirmed by a number of studies (Gabrielsen et al., 2016; Zhong et al., 2018; Zhang et al., 2019). Hundreds of risk susceptibility genes related to AIDs have been identified through genome-wide scans and large-scale single-nucleotide polymorphism (SNP) association studies (Lessard et al., 2012; Kochi, 2016). Many studies have confirmed the genetic background of AIDs.

Inflammasomes are highly conserved pattern recognition receptors in the cytoplasm of cells and are an important part of the innate immune system (Evavold and Kagan, 2019). The most characteristic inflammasome is the nucleotide oligomerization domain-like receptor (NLR) family pyrin domain-containing 3 (NLRP3) inflammasome. NLRP3, also known as CIAS1/NALP3/cryopyrin, is a well-known member of NLRs (de Torre-Minguela et al., 2017). NLRP3 is mainly expressed in macrophages and dendritic cells, and its main function is inflammasome assembly. The NLRP3 inflammasome is a multi-protein complex composed of NLRP3, apoptosis-associated speck-like protein (ASC), and proinflammatory caspase-1 (de Torre-Minguela et al., 2017; Wang et al., 2020). The NLRP3 inflammasome can recognize pathogen- or danger-associated molecular patterns. Once NLRP3 is activated, the adaptor protein ASC can be recruited, resulting in the cleavage of pro-caspase-1 into caspase-1. Activated caspase-1 processes pro-IL-1β and pro-IL-18 into the mature forms and secretes them outside the cell. At the same time, the activated caspase-1 fragment can induce cell membrane perforation, cell rupture, and cell pyroptosis (Fusco et al., 2020). Growing evidences indicate that NLRP3 inflammasome plays a key role in various AIDs (Shen et al., 2018; Zhao et al., 2018; Alves da Cruz et al., 2020).

The human NLRP3 gene is about 32.9-kb long. It is located on chromosome 1q44 and consists of nine exons (Villani et al., 2009). Single-nucleotide polymorphisms in the NLRP3 gene may lead to changes in its function, increasing the activation of inflammasomes and the levels of IL-1β. So far, about 60 SNPs in the NLRP3 gene have been identified. Among them, the most common polymorphisms are rs35829419 (Q705K), rs10754558, rs4612666, rs4925648, and rs10925019 (Zhang et al., 2011). This study focuses on the two most-studied polymorphic sites of NLRP3, including rs35829419, a gain-of-function polymorphism associated with pro-inflammatory phenotype (Verma et al., 2012), and rs10754558, which is located in the 3′-untranslated region (3′-UTR) of the NLRP3 gene and has a certain impact on the stability of NLRP3 mRNA (Hitomi et al., 2009). Many studies have explored the association between NLRP3 rs35829419/rs10754558 polymorphisms and AIDs, including multiple sclerosis (MS) (Imani et al., 2018), rheumatoid arthritis (RA) (Kastbom et al., 2010; Ben et al., 2012; Jenko et al., 2016; Addobbati et al., 2018), psoriatic arthritis (PsA) (Juneblad et al., 2020), celiac disease (CD) (Pontillo et al., 2011), type 1 diabetes (T1D) (Pontillo et al., 2010; Smigoc et al., 2019), myasthenia gravis (MG) (Agah et al., 2021), and systemic lupus erythematosus (SLE) (Pontillo et al., 2012; Su et al., 2020). Some studies show that these NLRP3 polymorphism sites are related to the risk of certain AIDs, but other studies suggest that NLRP3 gene polymorphism at these sites exhibit a disease protection effect. Some studies also indicate that NLRP3 rs35829419/rs10754558 polymorphisms have nothing to do with the risk of AIDs. Therefore, the views about the association between NLRP3 gene polymorphisms at these two loci and AIDs remain contradictory and inconclusive, partially because of the relatively small sample size, low statistical power, and/or disease heterogeneity in these studies. Hence, to resolve this issue, we conducted a comprehensive meta-analysis to evaluate whether NLRP3 rs35829419 C/A and rs10754558 C/G polymorphisms are associated with AIDs.

## Materials and Methods

### Publication Search

To explore the association of the NLRP3 rs35829419 C/A and rs10754558 C/G polymorphisms with AIDs, we searched the literature in PUBMED and Web of Science on February 1, 2021. The following keywords and medical subject heading terms were used for the search: (“NLRP3” or “CIAS1” or “NALP3” or “Cryopyrin” or “rs35829419” or “rs10754558”) and (“polymorphism” or “SNP” or “variation” or “mutation”). All eligible studies were retrieved, and their references were checked and manually searched for finding other relevant articles. The related literature published in previous meta-analyses was also searched to identify other special publications. Only articles published in English and with human subjects were selected for this meta-analysis.

### Inclusion and Exclusion Criteria

Studies included in this meta-analysis had to meet all four criteria: (a) evaluation of association of rs35829419 or rs10754558 with AIDs risk; (b) original study and case-control design; (c) provision of sufficient genotype or allele data in cases and controls to calculate odds ratio (OR) and 95% confidence intervals (CIs); (d) full-text available. The definitions of patients with different types of AIDs in the selected literature followed internationally recognized guidelines such as the European League Against Rheumatism (EULAR)/American College of Rheumatology (ACR) (Wayant et al., 2020; Fanouriakis et al., 2021; Kostine et al., 2021), the American Diabetes Association (American Diabetes Association, 2020), the American College of Gastroenterology (Lacy et al., 2021), the Myasthenia Gravis Foundation of America (Narayanaswami et al., 2021), and the International Panel on Diagnosis of Multiple Sclerosis (Thompson et al., 2018). Accordingly, the exclusion criteria were as follows: (a) diseases not clearly regarded as AIDs; (b) not a case-control study; (c) no available genotype frequency; and (d) review, meta-analysis, case report, and comments. To ensure the accuracy of the included studies, all evaluations were independently performed by two separate researchers.

### Data Extraction

All the data from all eligible studies were independently extracted by two investigators using the selection criteria above and checked by other authors. Any disagreement was resolved by discussion. We collected the following information from each study: ages and genders of research subjects, name of first author, year of publication, country, patient ethnicity, disease type, genotyping method, and total numbers and genotype/allele frequency distributions of both cases and controls.

### Quality Assessment

We used the Newcastle–Ottawa Quality Assessment Scale to evaluate the quality score of the included studies. High-quality research was defined with a score ≥7.

### Statistical Analysis

Hardy–Weinberg equilibrium (HWE) was tested by the Chi-square test. The crude OR and its 95% CIs in each case-control study were used to evaluate the strength of association between NLRP3 rs35829419 or rs10754558 polymorphisms and the risk of AIDs. The statistical significance of the pooled OR was determined by the Z-test, and a *p*-value of <0.05 was considered statistically significant. The results were summarized in the following models: an allele model (A vs. C for rs35829419; G vs. C for rs10754558), a dominant model (AA/AC vs. CC for rs35829419; GG/GC vs. CC for rs10754558), a recessive model (AA vs. AC/CC for rs35829419; GG vs. GC/CC for rs10754558), a homozygote co-dominant model (AA vs. CC for rs35829419; GG vs. CC for rs10754558), and a heterozygote co-dominant model (AC vs. CC for rs35829419; GC vs. CC for rs10754558), respectively. Subgroup analyses were conducted by ethnicity and disease type. Heterogeneity analysis was examined by the Chi-square based on Q-test. If the result of this heterogeneity test was *p* < 0.05, then the pooled OR was analyzed using a random-effects model (the DerSimonian and Laird method) to evaluate the association of NLRP3 Single-nucleotide polymorphisms with AIDs. Otherwise, a fixed-effects model (the Mantel–Haenszel method) was selected. Besides, in order to quantitatively estimate heterogeneity, we used another measure, *I*^2^ = 100% × (Q – *df*)/Q. The *I*^2^ statistic estimated the degree of inconsistency in the studies (*I*^2^ < 25% no heterogeneity; *I*^2^ = 25–50% moderate heterogeneity; *I*^2^ > 50% extreme heterogeneity). Publication bias was investigated using Begg's funnel plot and Eegg's test. If the funnel plot was asymmetric, and the Eegg's result was *p* < 0.05, it was considered that there might be a publication bias. Meanwhile, we performed a sensitivity analysis in which one study was removed, and the rest were conducted to evaluate whether the results were affected statistically significantly. In this study, the meta-analysis was performed by using the software STATA version 14.0 (Stata Corporation, College Station, TX, USA).

## Results

### Baseline Characteristics of Included Studies

**Figure 1:**
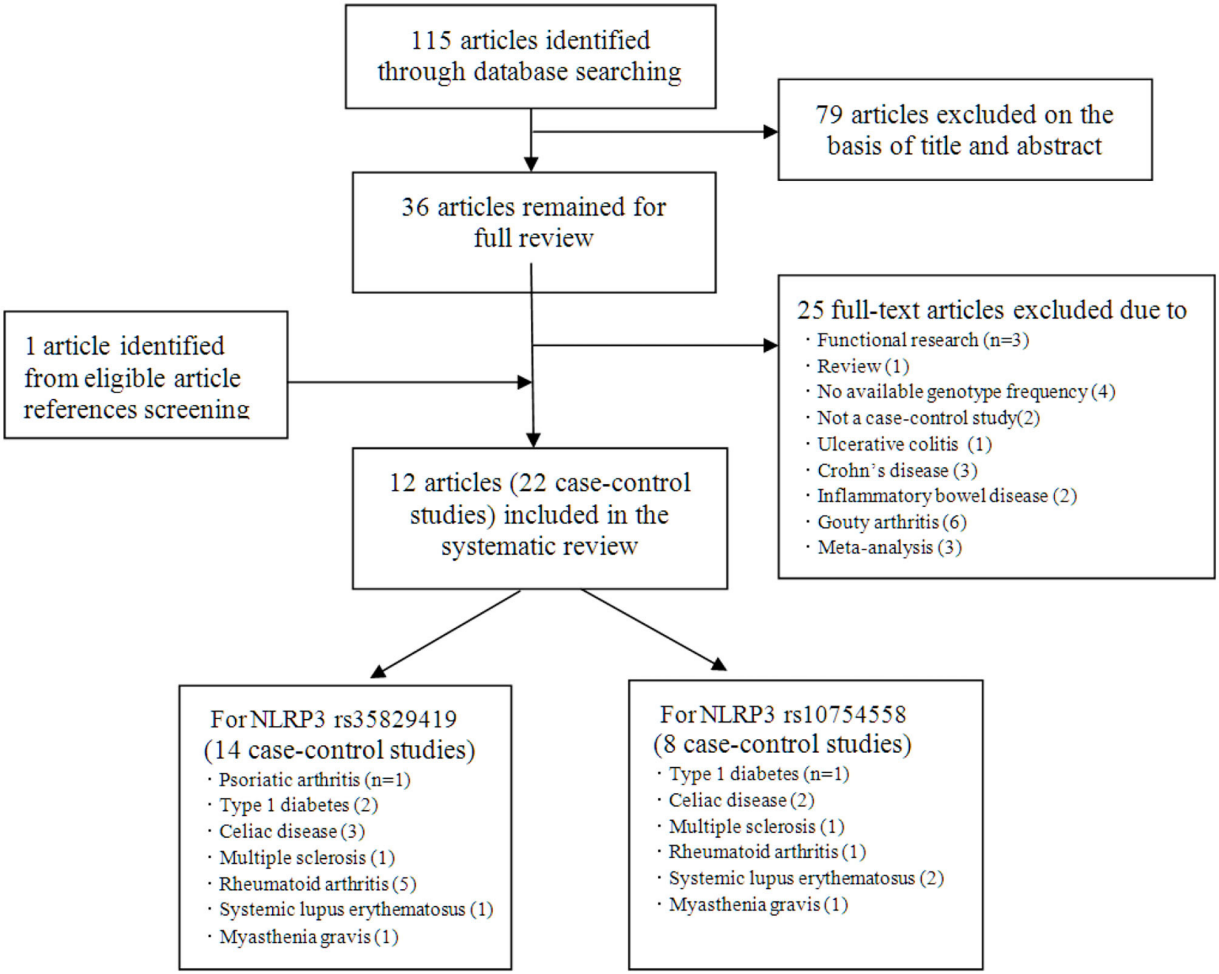
Flowchart of study selection.

Initially, 115 studies were identified by electronic searches. Among them, according to the title and abstract, a total of 79 non-inflammatory susceptibility studies were excluded. Finally, 36 full-text articles were included in our detailed analysis. After careful evaluation of the published literature based on the inclusion and exclusion criteria, we finally identified 12 articles to be included in this meta-analysis (Figure 1). For the NLRP3 rs35829419 polymorphic site, there were 11 articles in total. In these articles, sometimes one article involved several AIDs, and we regarded one of the AIDs as a case-control study. In the end, it included 14 case-control studies with 3,149 cases and 3,081 controls to assess the relationship between NLRP3 rs35829419 and AIDs. For the NLRP3 rs10754558 polymorphic site, there were a total of six articles. Eight case-control studies with 1,764 cases and 1,661 controls were included to evaluate the relationship between NLRP3 rs10754558 and AIDs. All studies were of case-control design and targeted at RA (*n* = 4), T1D (2), CD (3), SLE (2), psoriatic arthritis (PsA, 1), MS (1), and MG (1). All cases were patients with AIDs, and the controls were free of AIDs. In the included articles, although three articles were published by the same author, the research populations did not overlap with each other. The genotyping methods included fluorescence-based competitive allele-specific (KASPar), TaqMan, and multiplex ligation detection reaction (MLDR). The baseline characteristics of the eligible studies are summarized in Table 1.

**Table 1:** Baseline characteristics of eligible studies in all included studies.

**References**	**Country**	**Disease type**	**Genotyping method**	**Gene type**	**Age (years)**		**Gender (M/F)**		**Numbers**	
					**Cases**	**Controls**	**Cases**	**Controls**	**Cases**	**Controls**
Juneblad et al., 2020	Sweden	PsA	Taqman	rs35829419	59.4 ± 12.4	–	337/387	153/434	724	587
Smigoc et al., 2019	Slovenia	T1D	KASPar	rs35829419	7.8(3.6–11.4)	–	32/33	–	65	126
	Slovenia	CD	KASPar	rs35829419	4.8 (2.3–9.5)	–	23/44	–	67	126
Pontillo et al., 2010	Brazil	T1D	Taqman	rs35829419	13.2 ± 4.1	7.1 ± 4.8	94/102	90/102	196	192
	Brazil	T1D	Taqman	rs10754558	13.2 ± 4.1	7.1 ± 4.8	94/102	90/102	196	192
	Brazil	CD	Taqman	rs35829419	10 ± 4	7.1 ± 4.8	21/38	90/102	59	192
	Brazil	CD	Taqman	rs10754558	10 ± 4	7.1 ± 4.8	21/38	90/102	59	192
Imani et al., 2018	Iran	MS	Taqman	rs35829419	34.2 ± 9.2	32.5 ± 6.5	62/88	40/60	150	100
	Iran	MS	Taqman	rs10754558	34.2 ± 9.2	32.5 ± 6.5	62/88	40/60	150	100
Addobbati et al., 2018	Brazil	RA	Taqman	rs35829419	–	–	13/205	109/198	218	307
	Brazil	RA	Taqman	rs10754558	–	–	13/205	109/198	218	307
Jenko et al., 2016	Slovenia	RA	KASPar	rs35829419	56.9 (45.5–65.3)	53 (47–60)	26/102	–	128	122
Ben et al., 2012	France	RA	Taqman	rs35829419	32 ± 10	–	13/87	–	100	100
	Tunisia	RA	Taqman	rs35829419	39.5 ± 20	42.8	23/118	58/133	141	191
Kastbom et al., 2010	Sweden	RA	Taqman	rs35829419	54.4 ± 14.1	–	180/380	–	560	568
Pontillo et al., 2011	Italy	CD	Taqman	rs35829419	12.67 ± 11.25	42 ± 7	197/307	118/138	504	256
	Italy	CD	Taqman	rs10754558	12.67 ± 11.25	42 ± 7	197/307	118/138	504	256
Su et al., 2020	China	SLE	MLDR	rs10754558	36.33 ± 12.99	37.21 ± 12.16	41/359	55/345	400	400
Pontillo et al., 2012	Brazil	SLE	Taqman	rs35829419	37 ± 11.9	34.7 ± 11.69	8/136	83/75	144	158
	Brazil	SLE	Taqman	rs10754558	37 ± 11.9	34.7 ± 11.69	8/136	83/75	144	158
Agah et al., 2021	Iran	MG	Taqman	rs35829419	45.6 ± 14.9	–	34/59	24/32	93	56
	Iran	MG	Taqman	rs10754558	45.6 ± 14.9	–	34/59	24/32	93	56

*M, male; F, female; PsA, psoriatic arthritis; T1D, type 1 diabetes; CD, celiac disease; KASPar, fluorescence-based competitive allele-specific; MS, multiple sclerosis; RA, rheumatoid arthritis; SLE, systemic lupus erythematosus; MLDR, multiplex ligation detection reaction; MG, myasthenia gravis*.

### Allele and Genotype Frequencies of Included Studies

Details of allele and genotype frequencies are shown in Table 2. In several articles, the data in the control group deviated from the HWE (*p* < 0.05). Among these articles, three articles came from the Latin American populations, six articles came from the European populations, three articles came from the Arab populations, and one article came from the Asian populations. Ethnicity-subgroup meta-analysis was performed to determine the association of the NLRP3 rs35829419 and rs10754558 polymorphisms with AIDs in different races. Furthermore, disease-subgroup meta-analysis was conducted to explore the association of the NLRP3 rs35829419 and rs10754558 polymorphisms with PsA, T1D, CD, MS, RA, SLE, and MG.

**Table 2:** Data of allele and genotype frequencies in all included studies.

**References**	**Ethnicity**	**Disease type**	**Gene type**	**Cases**					**Controls**					**HWE**
				**C/C**	**A/G**	**CC/CC**	**CA/CG**	**AA/GG**	**C/C**	**A/G**	**CC/CC**	**CA/CG**	**AA/GG**	
Juneblad et al., 2020	European	PsA	rs35829419	1,338	98	622	94	2	1,088	84	505	78	3	0.995
Smigoc et al., 2019	European	T1D	rs35829419	123	7	59	5	1	236	16	111	14	1	0.461
	European	CD	rs35829419	125	9	58	9	0	236	16	111	14	1	0.461
Pontillo et al., 2010	Latin American	T1D	rs35829419	388	4	192	4	0	380	4	188	4	0	0.884
	Latin American	T1D	rs10754558	264	128	94	76	26	219	165	69	81	42	0.054
	Latin American	CD	rs35829419	109	9	50	9	0	380	4	188	4	0	0.884
	Latin American	CD	rs10754558	80	38	27	26	6	218	166	67	84	41	0.132
Imani et al., 2018	Arab	MS	rs35829419	274	26	126	22	2	184	16	88	8	4	*p* < 0.05
	Arab	MS	rs10754558	162	138	47	68	35	100	100	19	62	19	0.016
Addobbati et al., 2018	Latin American	RA	rs35829419	427	9	209	9	0	585	23	284	17	3	*p* < 0.05
	Latin American	RA	rs10754558	288	144	101	86	29	339	269	97	145	62	0.562
Jenko et al., 2016	European	RA	rs35829419	242	14	114	14	0	224	20	105	14	3	0.009
Ben et al., 2012	European	RA	rs35829419	192	8	92	8	0	192	8	93	6	1	0.029
	Arab	RA	rs35829419	274	8	133	8	0	365	17	174	17	0	0.520
Kastbom et al., 2010	European	RA	rs35829419	1,023	63	481	61	1	1,056	80	490	76	2	0.601
Pontillo et al., 2011	European	CD	rs35829419	974	34	470	34	0	482	30	226	30	0	0.319
	European	CD	rs10754558	561	447	154	253	97	280	232	79	122	55	0.539
Su et al., 2020	Asian	SLE	rs10754558	470	330	142	186	72	441	359	134	173	93	0.012
Pontillo et al., 2012	Latin American	SLE	rs35829419	279	9	135	9	0	303	15	146	11	2	0.004
	Latin American	SLE	rs10754558	175	113	54	67	23	166	150	42	82	34	0.609
Agah et al., 2021	Arab	MG	rs35829419	173	13	82	9	2	107	3	52	3	0	0.835
	Arab	MG	rs10754558	101	83	26	49	17	56	56	11	34	11	0.109

*HWE, Hardy–Weinberg equilibrium; PsA, psoriatic arthritis; T1D, type 1 diabetes; CD, celiac disease; MS, multiple sclerosis; RA, rheumatoid arthritis; SLE, systemic lupus erythematosus; MG, myasthenia gravis*.

### Meta-Analysis for Association Between Nucleotide Oligomerization Domain-Like Receptor Family Pyrin Domain-Containing 3 rs35829419 Polymorphisms and Risks of Autoimmune Diseases

In the included studies, we observed significant heterogeneity in some models, so the random effects model was applied, and the M–H fixed effects model was used in the other models. In all, when all eligible studies were pooled into the meta-analyses, we found that no significant AIDs risk was associated with the NLRP3 rs35829419 polymorphism in the allelic model (A vs. C: OR = 0.89, 95% CI = 0.69–1.14), the heterozygote co-dominant model (AC vs. CC: OR = 1.00, 95% CI = 0.77–1.30), or the dominant model (AA/AC vs. CC: OR = 0.93, 95% CI = 0.71–1.20). However, a protected effect was found in the recessive model (AA vs. AC/CC: OR = 0.45, 95% CI = 0.22–0.92, *p* = 0.029) and the homozygote co-dominant model (AA vs. CC: OR = 0.45, 95% CI = 0.22–0.93, *p* = 0.030). In the subgroup analysis by ethnicity, statistically slightly decreased AIDs risks were found among European populations in the allelic model (A vs. C: OR = 0.83, 95% CI = 0.69–0.99, *p* = 0.043). However, in other genetic models, no association between NLRP3 rs35829419 polymorphism and AIDs susceptibility was observed. More importantly, among the Latin American and Arabian populations, we also found no association between NLRP3 rs35829419 polymorphism and AIDs risk in any genetic model. In the subgroup of disease type, there were no significantly decreased or increased risks of PsA, T1D, CD, MS, SLE, and MG in any genetic model. However, in the RA group, remarkably decreased risks were found in the allelic model (A vs. C: OR = 0.74, 95% CI = 0.57–0.96, *p* = 0.024) and the homozygote co-dominant model (AA vs. CC: OR = 0.27, 95% CI = 0.08–0.97, *p* = 0.045). No significant associations were found in any other genetic model. Details of the meta-analysis are shown in Table 3 and Supplementary Table 1.

**Table 3:** Summary of odd ratios (OR) and 95% CIs of NLRP3 rs35829419 polymorphism and autoimmune diseases (AIDs) susceptibility for various comparisons.

**Stratification**	***N***	**AA vs. CC**		**AC vs. CC**		**AA/AC vs. CC**		**AA vs. AC/CC**		**A vs. C**	
		**OR (95%CIs)**	***P_***h***_***	**OR (95%CIs)**	***P_***h***_***	**OR (95%CIs)**	***P_***h***_***	**OR (95%CIs)**	***P_***h***_***	**OR (95%CIs)**	***P_***h***_***
Total	14	0.45 (0.22, 0.93)	0.948	1.00 (0.77, 1.30)	0.043	0.93 (0.71, 1.20)	0.036	0.45 (0.22, 0.92)	0.948	0.89 (0.69, 1.14)	0.038
**Ethnicity**											
European	7	0.47 (0.17, 1.29)	0.882	0.86 (0.70, 1.04)	0.520	0.84 (0.69, 1.02)	0.625	0.48 (0.17, 1.30)	0.877	0.83 (0.69, 0.99)	0.673
Latin American	4	0.20 (0.02, 1.71)	0.960	1.45 (0.51, 4.14)	0.008	1.32 (0.44, 4.00)	0.004	0.21 (0.02, 1.73)	0.963	1.21 (0.39, 3.72)	0.002
Arab	3	0.62 (0.18, 2.09)	0.449	1.23 (0.77, 1.98)	0.242	1.09 (0.66, 1.78)	0.134	0.60 (0.18, 2.03)	0.449	1.06 (0.67, 1.69)	0.175
**Disease type**											
PsA	1	0.54 (0.09, 3.25)	NA	0.98 (0.71, 1.35)	NA	0.96 (0.70, 1.32)	NA	0.54 (0.09, 3.26)	NA	0.95 (0.70, 1.28)	NA
T1D	2	1.88 (0.12, 30.62)	NA	0.77 (0.33, 1.78)	0.675	0.82 (0.37, 1.84)	0.764	1.95 (0.12, 31.74)	NA	0.88 (0.41, 1.88)	0.856
CD	3	0.64 (0.03, 15.84)	NA	1.63 (0.39, 6.82)	< 0.001	1.59 (0.39, 6.55)	<0.001	0.62 (0.02, 15.42)	NA	1.52 (0.40, 5.78)	<0.001
MS	1	0.35 (0.06, 1.95)	NA	1.92 (0.82, 4.51)	NA	1.40 (0.66, 2.94)	NA	0.32 (0.06, 1.81)	NA	1.09 (0.57, 2.09)	NA
RA	5	0.27 (0.08, 0.97)	0.962	0.84 (0.65, 1.10)	0.921	0.76 (0.58, 1.00)	0.827	0.28 (0.08, 1.00)	0.957	0.74 (0.57, 0.96)	0.813
SLE	1	0.22 (0.01, 4.54)	NA	0.88 (0.36, 2.20)	NA	0.75 (0.31, 1.81)	NA	0.22 (0.01, 4.58)	NA	0.65 (0.28, 1.51)	NA
MG	1	3.18 (0.15, 67.59)	NA	1.90 (0.49, 7.35)	NA	2.33 (0.62, 8.73)	NA	3.03 (0.14, 64.33)	NA	2.68 (0.75, 9.62)	NA

*N, number of studies involved; P_h_, p-value of Q-test for heterogeneity test; OR, odds ratios; NA, not available; PsA, psoriatic arthritis; T1D, type 1 diabetes; CD, celiac disease; MS, multiple sclerosis; RA, rheumatoid arthritis; SLE, systemic lupus erythematosus; MG, myasthenia gravis*.

### Meta-Analysis for Association Between Nucleotide Oligomerization Domain-Like Receptor Family Pyrin Domain-Containing 3 rs10754558 Polymorphisms and Autoimmune Diseases Risks

Since there was no significant heterogeneity in the following eight articles, the M–H fixed effects model was used for meta-analysis. On the whole, NLRP3 rs10754558 polymorphism contributed to reducing the risk of AIDs in all genetic models, including the allelic model (G vs. C: OR = 0.78, 95% CI = 0.71–0.87, *p* < 0.001), the homozygote co-dominant model (GG vs. CC: OR = 0.63, 95% CI = 0.51–0.77, *p* < 0.001), the heterozygote co-dominant model (GC vs. CC: OR = 0.78, 95% CI = 0.66–0.91, *p*=0.002), the dominant model (GG/GC vs. CC: OR = 0.73, 95% CI = 0.63–0.84, *p* < 0.001), and the recessive model (GG vs. GC/CC: OR = 0.73, 95% CI = 0.62-0.88, *p* = 0.001). The mutation of allele from C to G can play a protective role against AIDs. Besides, in the subgroup analysis by ethnicity, significantly decreased AIDs risks were also observed among the Latin American populations in all genetic models. Among the Arabian populations, we only observed a significant reduction in the risk of AIDs in the dominant model and the heterozygote co-dominant model. In other genetic models, no association between NLRP3 rs10754558 polymorphism and AIDs susceptibility was observed. However, among European and Asian populations, we found no relationship between NLRP3 rs10754558 polymorphism and AIDs risk in any genetic model. In the subgroup of disease type, no significantly decreased or increased risks of CD and MG were found in any genetic model. However, in the RA group, remarkably decreased risks were found in all genetic models. In the T1D group, other genetic models in addition to the heterozygote co-dominant model showed that NLRP3 rs10754558 polymorphism was associated with a decreased risk of T1D. In the SLE group, the results of the allelic model, the homozygote co-dominant model, and the recessive model indicate that NLRP3 rs10754558 polymorphism can help reduce the risk of SLE. In the MS group, the results of the heterozygote co-dominant model and the dominant model also show the protective effect of NLRP3 rs10754558 polymorphism. No significant associations were found in any other genetic models. Details of the meta-analysis are shown in Table 4 and Supplementary Table 2.

**Table 4:** Summary of OR and 95% CIs of NLRP3 rs10754558 polymorphism and AIDs susceptibility for various comparisons.

**Stratification**	***N***	**GG vs. CC**		**GC vs. CC**		**GG/GC vs. CC**		**GG vs. GC/CC**		**G vs. C**	
		**OR (95% CIs)**	***P_***h***_***	**OR (95% CIs)**	***P_***h***_***	**OR (95% CIs)**	***P_***h***_***	**OR (95% CIs)**	***P_***h***_***	**OR (95% CIs)**	***P_***h***_***
Total	8	0.63 (0.51, 0.77)	0.345	0.78 (0.66, 0.91)	0.082	0.73 (0.63, 0.84)	0.096	0.73 (0.62, 0.88)	0.370	0.78 (0.71, 0.87)	0.159
**Ethnicity**											
European	1	0.90 (0.59, 1.39)	NA	1.06 (0.75, 1.50)	NA	1.01 (0.73, 1.41)	NA	0.87 (0.60, 1.26)	NA	0.96 (0.78, 1.19)	NA
Latin American	4	0.45 (0.33, 0.62)	0.941	0.64 (0.51, 0.81)	0.854	0.58 (0.47, 0.72)	0.945	0.58 (0.44, 0.78)	0.817	0.65 (0.56, 0.76)	0.935
Arab	2	0.71 (0.38, 1.32)	0.844	0.50 (0.30, 0.82)	0.550	0.55 (0.34, 0.89)	0.713	1.15 (0.70, 1.91)	0.531	0.84 (0.63, 1.12)	0.905
Asian	1	0.73 (0.50, 1.08)	NA	1.01 (0.74, 1.39)	NA	0.92 (0.68, 1.23)	NA	0.72 (0.51, 1.02)	NA	0.86 (0.71, 1.05)	NA
**Disease type**											
T1D	1	0.45 (0.25, 0.81)	NA	0.69 (0.44, 1.07)	NA	0.61 (0.41, 0.91)	NA	0.55 (0.32, 0.93)	NA	0.64 (0.48, 0.86)	NA
CD	2	0.76 (0.52, 1.12)	0.090	0.99 (0.73, 1.34)	0.373	0.91(0.68, 1.21)	0.175	0.77 (0.55, 1.08)	0.141	0.88 (0.73, 1.07)	0.08
MS	1	0.74 (0.34, 1.61)	NA	0.44 (0.24, 0.84)	NA	0.51 (0.28, 0.94)	NA	1.30 (0.69, 2.43)	NA	0.85 (0.60, 1.22)	NA
RA	1	0.45 (0.27, 0.76)	NA	0.57 (0.39, 0.84)	NA	0.53 (0.37, 0.76)	NA	0.61 (0.37, 0.98)	NA	0.63 (0.49, 0.81)	NA
SLE	2	0.67 (0.48, 0.94)	0.403	0.89 (0.68, 1.17)	0.129	0.82 (0.64, 1.05)	0.151	0.72 (0.53, 0.96)	0.898	0.82 (0.69, 0.97)	0.331
MG	1	0.65 (0.23, 1.84)	NA	0.78 (0.66, 0.91)	NA	0.62 (0.28, 1.38)	NA	0.93 (0.40, 2.16)	NA	0.82 (0.51, 1.32)	NA

*N, number of studies involved; P_h_, p-value of Q-test for heterogeneity test; OR, odds ratios; NA, not available; T1D, type 1 diabetes; CD, celiac disease; MS, multiple sclerosis; RA, rheumatoid arthritis; SLE, systemic lupus erythematosus; MG, myasthenia gravis*.

### Risk of Bias Across Studies and Within Studies

**Figure 2:**
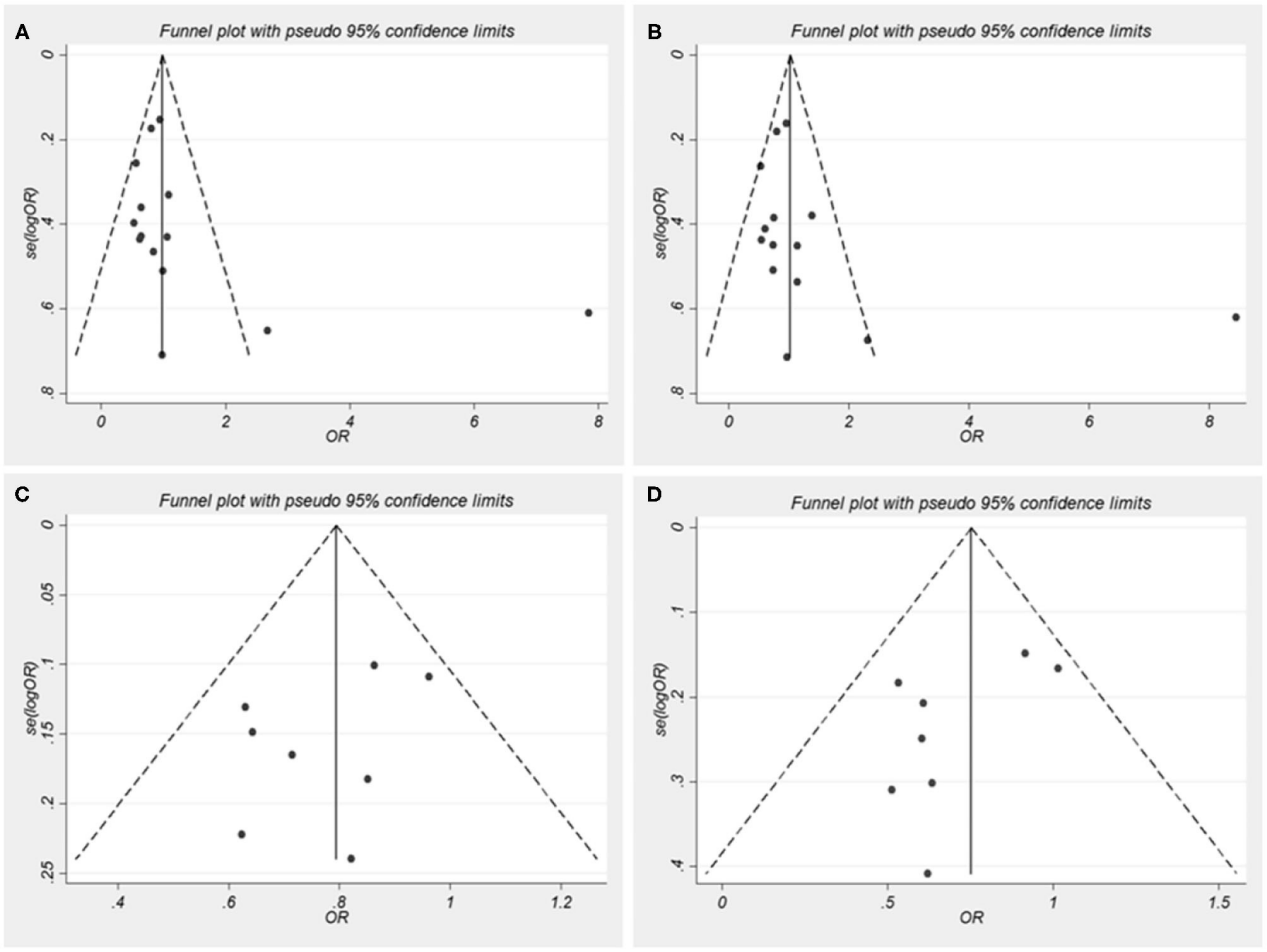
Funnel plots for nucleotide oligomerization domain-like receptor family pyrin domain-containing 3 (NLRP3) single nucleotide polymorphisms (SNPs) and autoimmune diseases (AIDs) risk. OR, odds ratios. **(A)** A vs. C for rs35829419; **(B)** AA/AC vs. CC for rs35829419; **(C)** G vs. C for rs10754558; **(D)** GG/GC vs. CC for rs10754558.

Begger's funnel plot and Egger's test were performed to assess the publication bias of the literature. In all comparative models, the shape of the funnel plots was asymmetrical, which shows some evidence of publication bias (Figure 2). However, Egger's test can provide enough statistical evidence about funnel plot asymmetry. Results do not show any evidence of publication bias (A vs. C: *t* = 1.36, *p* = 0.199; AA/AC vs. CC: *t* = 1.36, *p* = 0.199; G vs. C: *t* = −1.25, *p* = 0.259; GG/GC vs. CC: *t* = −1.81, *p* = 0.120; Figure 2). In addition, individual studies were assessed for bias using the Newcastle–Ottawa Scale for case-control studies (Supplementary Table 3). Two studies scored 9 points, 13 studies 8 points, and the rest scored 7 points out of a total 10 points.

### Forest Plot and Sensitivity Analysis

**Figure 3:**
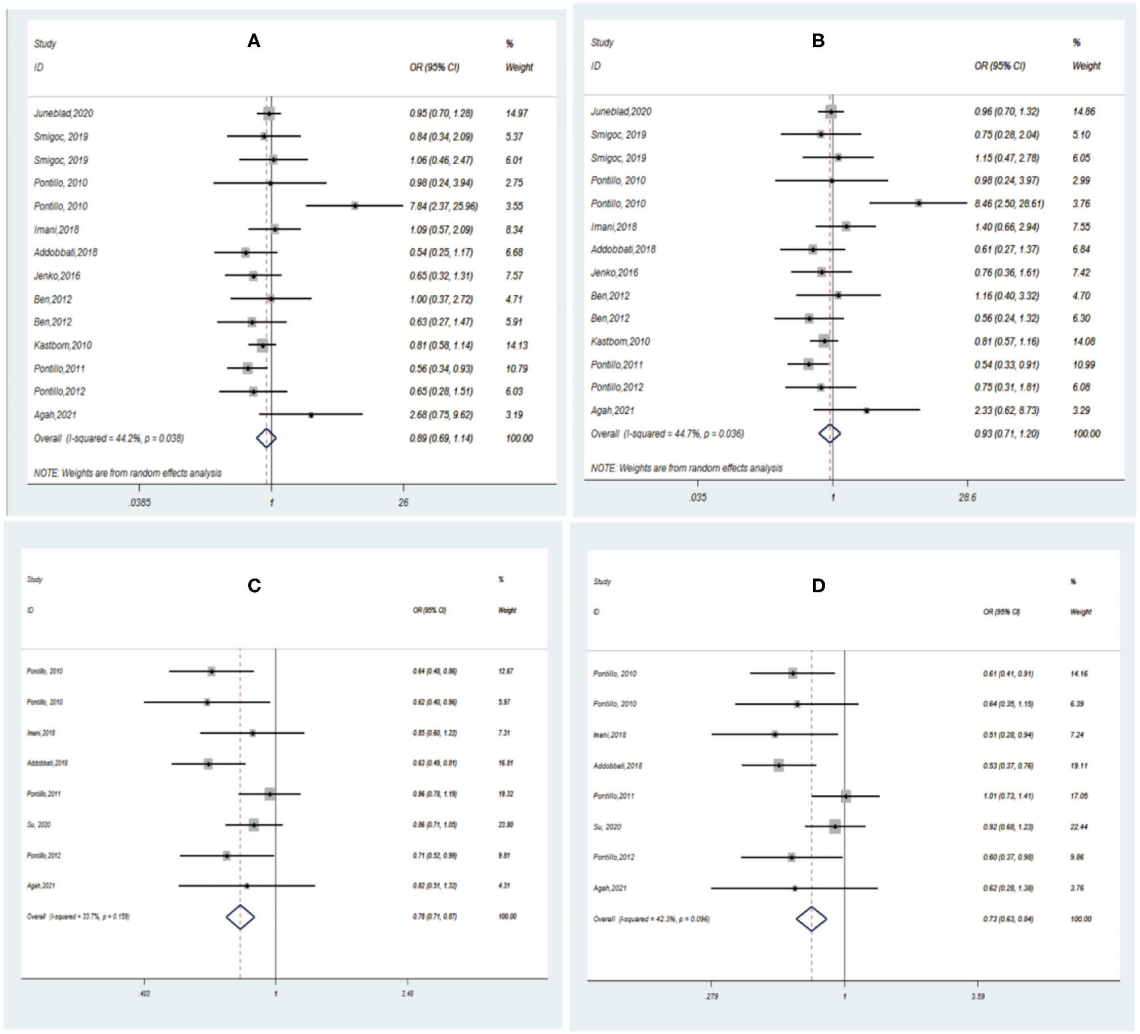
Forest plot of AIDs risk associated with NLRP3 SNPs under the allele/dominant model contrast. **(A)** A vs. C for rs35829419; **(B)** AA/AC vs. CC for rs35829419; **(C)** G vs. C for rs10754558; **(D)** GG/GC vs. CC for rs10754558.

**Figure 4:**
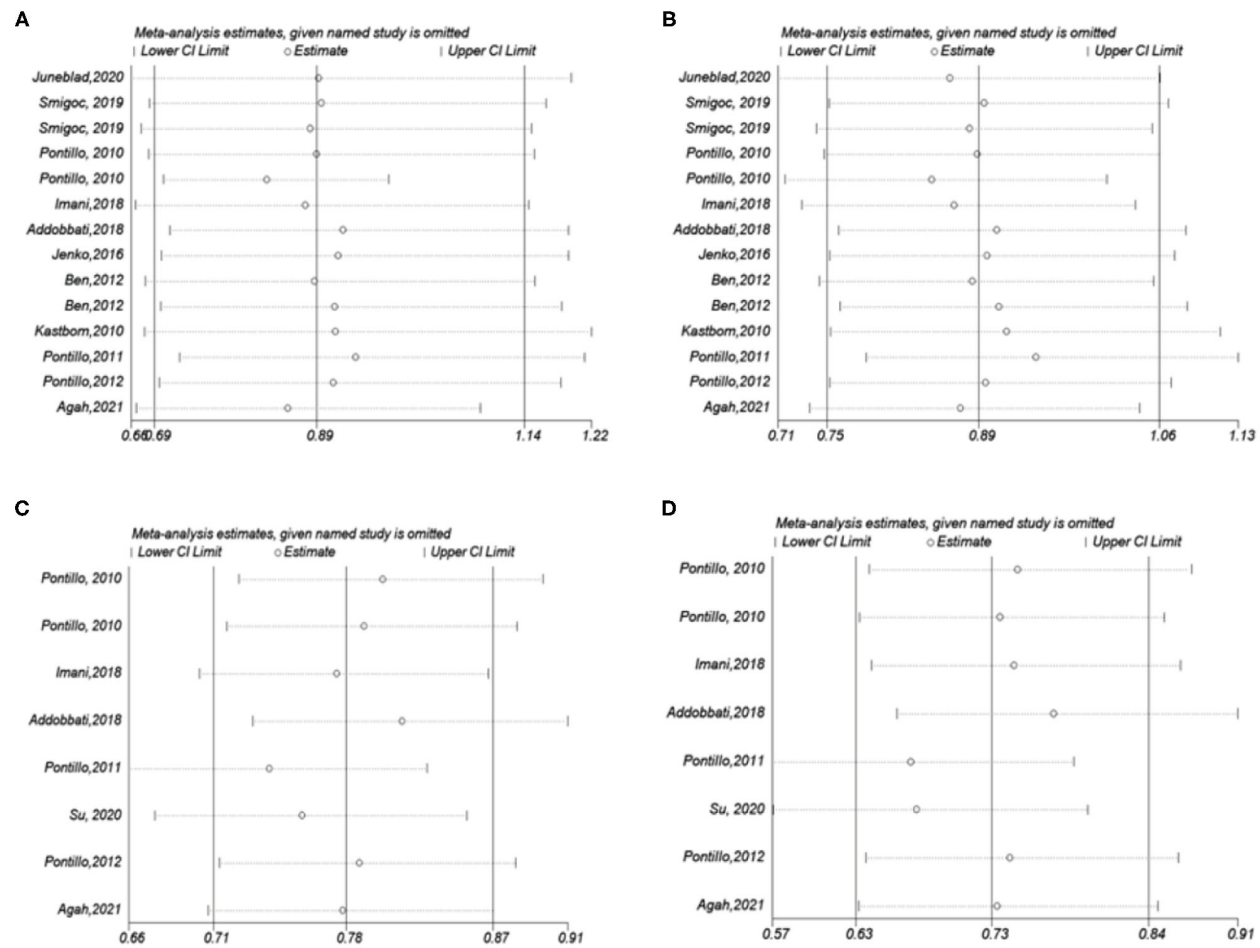
Influence analysis for NLRP3 SNPs in the overall meta-analysis. The middle vertical axis indicates the overall OR and the other two vertical axes indicate the 95% CI. **(A)** A vs. C for rs35829419; **(B)** AA/AC vs. CC for rs35829419; **(C)** G vs. C for rs10754558; **(D)** GG/GC vs. CC for rs10754558.

Pooling the eligible studies, we found no association of NLRP3 rs35829419 with AIDs risk in the allele/dominant model (*p* = 0.339/*p* = 0.565, Figures 3A,B). However, we found a significant association between NLRP3 rs10754558 and AIDs risk in the allele/dominance model, and the G allele showed a protective effect (*p* < 0.001/*p* < 0.001, Figures 3C,D). In addition, sensitivity analysis was conducted to assess whether each eligible study affected the final results. After sequential removal of each study, we found the pooled OR was not influenced by any single study (Figure 4).

## Discussion

Single-nucleotide polymorphisms of DNA sequence are caused by nucleotide variation at the chromosome genome level and are among the important causes for drug responsiveness, disease susceptibility, and ethnic difference (Shastry, 2003). Recently, more researchers are devoted to revealing the relationship between NLRP3 SNPs and the susceptibility of inflammatory diseases. We systematically analyzed the relationships of NLRP3 rs35829419 and rs10754558 polymorphisms with susceptibility to AIDs through the existing research, so as to clarify the contradictory results brought by individual research. Our meta-results show that the NLRP3 rs35829419 polymorphism is unrelated with risk of occurrence of AIDs. However, this polymorphism may reduce the risk of RA susceptibility. In addition, significant association was observed between the NLRP3 rs10754558 polymorphism and susceptibility to AIDs, which tended to have a protective effect. Among the different races, this association was more significant among the Latin Americans. Analysis by the types of diseases showed that NLRP3 rs10754558 polymorphism was significantly related to the susceptibility of T1D, SLE, and RA, but not to other types of diseases.

Compared with previous meta-analyses, the published articles chosen in our research were stricter and more comprehensive. We searched all articles concerning the relationship between the NLRP3 rs35829419/rs10754558 polymorphisms and AIDs susceptibility and published in recent years through several databases, and excluded those inflammatory diseases such as Crohn's disease, ulcerative colitis, primary gout, and atherosclerosis, which were not removed in other meta-analyses. Previous meta-analyses focused on the relationship between NLRP3 polymorphisms and all inflammatory diseases, but our research was mainly concerned with the relationship between NLRP3 polymorphisms and AIDs. Finally, 12 qualified case–control studies were chosen in our research.

The NLRP3 rs35829419 variant is a C>A polymorphism located in exon 3 of its gene (Theodoropoulou et al., 2020). Considering that this variant is a gain-of-function mutation, it may lead to excessive production of IL-1β and IL-18. Therefore, researchers are keen to explore the role of NLRP3 rs35829419 in a variety of inflammatory diseases. However, current studies show it may have a different relationship with AIDs. Reportedly, the minor A allele of NLRP3 rs35829419 polymorphism seemingly exerts a protective role against the development of CD (Pontillo et al., 2011). However, another article about CD demonstrates that the NLRP3 rs35829419 A allele and especially the CA genotype seem to confer a higher risk for developing CD (Pontillo et al., 2010). Moreover, more evidence shows that NLRP3 rs35829419 is unrelated to the occurrence of AIDs, such as RA, CD, MS, and SLE. In addition, a meta-analysis by Zhang et al. includes multiple human diseases and shows that the NLRP3 rs35829419 gene polymorphism is generally a disease susceptibility factor related to various diseases (Zhang et al., 2015). In our meta-analysis, except for the homozygote co-dominant and recessive models, we found no significant association between NLRP3 rs35829419 and the overall AIDs, suggesting that polymorphism of this site may not play a key role in AIDs development. In a subgroup analysis by ethnicity, the allelic model showed a slightly, but not significantly, reduced risk of AIDs in the European population. Our meta-analysis results are consistent with Lee, who did not observe an association between autoimmune or inflammatory diseases and NLRP3 rs35829419 (Lee and Bae, 2016). Stratification by ethnicity also showed no association in the European, Latin American, or Polynesian populations. After stratification by disease type, the allelic and homozygote co-dominant models show that NLRP3 rs35829419 is significantly correlated to the susceptibility of RA, and the carrying of A allele contributes to the reduction of risk. In contrast to our results, an earlier meta-analysis shows that NLRP3 rs35829419 is not associated with the susceptibility to RA (Yang et al., 2017). In other AIDs, we found no association between NLRP3 rs35829419 and risk of AIDs in any genetic model. Although our analysis included more research data on RA, we cannot completely deny the irrelevance of NLRP3 rs35829419 gene polymorphism with RA. In fact, more research is required to explore the relationship.

The NLRP3 rs10754558 variant is a C>G polymorphism located in the 3′-UTR. Mutations in the 3′-UTR of NLRP3 gene may alter the activity of the inflammasome pathway by affecting the stability of mRNA and ultimately regulate the production of inflammatory factors including IL-1β and IL-18 (Hitomi et al., 2009). Compared with the C allele, the G allele of NLRP3 rs10754558 contributes to improving the stability of its mRNA, and the more stable NLRP3 expression may have a protective effect on the occurrence of T1D (Pontillo et al., 2010). However, the mechanism behind this seemingly contradictory phenomenon is unknown. Whether this enhanced NLRP3 mRNA stability affects the activation of inflammasomes and subsequent IL-1β secretion remains unknown. In this meta-analysis, we determine that NLRP3 rs10754558 in all genetic models exhibits a protective effect on reducing the risks of AIDs. The subgroup analysis of ethnicity shows a significant association between the NLRP3 rs10754558 polymorphism and AIDs in the Latin Americans, but not in the European, Arabian, or Asian populations. Ethnic differences in the population may be one of the root causes of this phenomenon, as the frequency of NLRP3 polymorphism may be different in different ethnic groups. This finding is consistent with a previous meta-analysis that includes other inflammatory diseases in addition to some AIDs (Lee and Bae, 2016). Meta-analysis by disease type shows no association between the NLRP3 rs10754558 polymorphism and MS, CD, or MG. However, in a variety of genetic models, meta-analysis results indicate that NLRP3 rs10754558 polymorphism is related to the susceptibility to T1D, RA, and SLE. NLRP3 rs10754558 polymorphism shows different associations with different AIDs, which may be related to the heterogeneity of the disease. Therefore, for the NLRP3 rs10754558 variant, a further study to confirm its protective effect against AIDs and its detailed mechanisms in AIDs progress is warranted. We speculate that this may be the result of a combination of innate immunity, local inflammation, and systemic environment.

There are some limitations that we cannot avoid. First, although the results of publication bias in our study are not significant, publication bias may also be caused by some unpublished studies of negative results or some articles ignored during the literature search. Second, some included studies have relatively small sample sizes, which may lead to lower statistical power, especially in the stratified analysis. Third, during subgroup analyses based on ethnicity and disease types, the numbers of studies in some subgroups are too small, which may reduce our analytical power. Moreover, in some studies, the control population does not conform to HWE, which may influence the accuracy of results. In addition, we found that although the authors of the original literature included in this meta-analysis did not statistically analyze age factors between the case and control groups for CD and T1D, there appeared to be significant age differences between the two groups. Whether it affects the association between NLRP3 polymorphism and susceptibility to CD or T1D is unclear. Finally, the research objects of our meta-analysis mainly focus on AIDs, including systemic AIDs and organ-specific AIDs. Different types of AIDs may have different pathogenesis due to their different pathophysiology and disease severity. NLPR3 may play a different role in different types and severity of AIDs. We speculate that these factors may have some influence on the results of the meta-analysis of the association of NLPR3 gene polymorphisms in autoimmune diseases.

In conclusion, the NLRP3 rs35829419 polymorphism may not be related to the risk of AIDs. However, NLRP3 rs10754558 allele G may have a protective effect on the risk of AIDs, especially in the Latin American population. In addition, NLRP3 rs10754558 polymorphism is associated with the occurrence of various AIDs, such as SLE, RA, and T1D. Further clinical research and functional analysis are needed to explore whether NLRP3 rs10754558 affects the production of cytokines through the inflammasome pathway.

## Data Availability Statement

The original contributions presented in the study are included in the article/Supplementary Material, further inquiries can be directed to the corresponding author/s.

## Author Contributions

ZW conducted the statistical analysis and revised the manuscript. TL designed the study and wrote the manuscript. SW collected the data and all authors contributed to the completion of the final manuscript.

## Conflict of Interest

The authors declare that the research was conducted in the absence of any commercial or financial relationships that could be construed as a potential conflict of interest.
