# Supplementary material for: Association of NLRP3 rs35829419 and rs10754558 Polymorphisms With Risks of Autoimmune Diseases: A Systematic Review and Meta-Analysis
Source: Front Genet. 2021 Jul 22;12:690860. doi: 10.3389/fgene.2021.690860 (PMC8340881; doi:10.3389/fgene.2021.690860)
Supplement: Supplementary file 1 [file Table_1.docx]

**Table S1**. Summary of OR and 95%CIs of NLRP3 rs35829419 polymorphism and AIDs susceptibility for various comparisons

| Stratification | N | | | | AA vs CC | | | | AC vs CC | | AA/AC vs CC | | AA vs AC/CC | | A vs C |  |
| --- | --- | --- | --- | --- | --- | --- | --- | --- | --- | --- | --- | --- | --- | --- | --- | --- |
|  | |  | | OR (95%CIs) | | | *P* | | OR (95%CIs) | *P* | OR (95%CIs) | *P* | OR (95%CIs) | *P* | OR (95%CIs) | *P* |
| Total | 14 | | 0.45 (0.22, 0.93) | | | 0.030 | | 1.00 (0.77, 1.30) | | 0.982 | 0.93 (0.71, 1.20) | 0.565 | 0.45 (0.22, 0.92) | 0.029 | 0.89 (0.69, 1.14) | 0.339 |
| Ethnicity |  | |  | | |  | |  | |  |  |  |  |  |  |  |
| European | 7 | | 0.47 (0.17, 1.29) | | | 0.144 | | 0.86 (0.70, 1.04) | | 0.125 | 0.84 (0.69, 1.02) | 0.072 | 0.48 (0.17, 1.30) | 0.147 | 0.83 (0.69, 0.99) | 0.043 |
| Latin American | 4 | | 0.20 (0.02, 1.71) | | | 0.143 | | 1.45 (0.51, 4.14) | | 0.491 | 1.32 (0.44, 4.00) | 0.619 | 0.21 (0.02, 1.73) | 0.146 | 1.21 (0.39, 3.72) | 0.740 |
| Arab | 3 | | 0.62 (0.18, 2.09) | | | 0.438 | | 1.23 (0.77, 1.98) | | 0.386 | 1.09 (0.66, 1.78) | 0.737 | 0.60 (0.18, 2.03) | 0.410 | 1.06 (0.67, 1.69) | 0.791 |
| Disease type |  | |  | | |  | |  | |  |  |  |  |  |  |  |
| PsA | 1 | | 0.54 (0.09, 3.25) | | | 0.502 | | 0.98 (0.71, 1.35) | | 0.895 | 0.96 (0.70, 1.32) | 0.813 | 0.54 (0.09, 3.26) | 0.504 | 0.95 (0.70, 1.28) | 0.733 |
| T1D | 2 | | 1.88 (0.12, 30.62) | | | 0.657 | | 0.77 (0.33, 1.78) | | 0.538 | 0.82 (0.37, 1.84) | 0.631 | 1.95 (0.12, 31.74) | 0.638 | 0.88 (0.41, 1.88) | 0.739 |
| CD | 3 | | 0.64 (0.03, 15.84) | | | 0.782 | | 1.63 (0.39, 6.82) | | 0.503 | 1.59 (0.39, 6.55) | 0.521 | 0.62 (0.02, 15.42) | 0.770 | 1.52 (0.40, 5.78) | 0.541 |
| MS | 1 | | 0.35 (0.06, 1.95) | | | 0.230 | | 1.92 (0.82, 4.51) | | 0.134 | 1.40 (0.66, 2.94) | 0.379 | 0.32 (0.06, 1.81) | 0.199 | 1.09 (0.57, 2.09) | 0.792 |
| RA | 5 | | 0.27 (0.08, 0.97) | | | 0.045 | | 0.84 (0.65, 1.10) | | 0.213 | 0.76 (0.58, 1.00) | 0.053 | 0.28 (0.08, 1.00) | 0.049 | 0.74 (0.57, 0.96) | 0.024 |
| SLE | 1 | | 0.22 (0.01, 4.54) | | | 0.324 | | 0.88 (0.36, 2.20) | | 0.793 | 0.75 (0.31, 1.81) | 0.520 | 0.22 (0.01, 4.58) | 0.327 | 0.65 (0.28, 1.51) | 0.319 |
| MG | 1 | | 3.18 (0.15, 67.59) | | | 0.458 | | 1.90 (0.49, 7.35) | | 0.351 | 2.33 (0.62, 8.73) | 0.211 | 3.03 (0.14, 64.33) | 0.477 | 2.68 (0.75, 9.62) | 0.131 |

N indicates the number of studies involved; OR: odds ratios; *P*: *P*-value of Z-test for OR; PsA: psoriatic arthritis; T1D: type 1 diabetes; CD: celiac disease; MS: multiple sclerosis; RA: rheumatoid arthritis; SLE: systemic lupus erythematosus; MG: myasthenia gravis
