# Supplementary material for: Association of NLRP3 rs35829419 and rs10754558 Polymorphisms With Risks of Autoimmune Diseases: A Systematic Review and Meta-Analysis
Source: Front Genet. 2021 Jul 22;12:690860. doi: 10.3389/fgene.2021.690860 (PMC8340881; doi:10.3389/fgene.2021.690860)
Supplement: Supplementary file 2 [file Table_2.docx]

**Table S2**. Summary of OR and 95%CIs of NLRP3 rs10754558 polymorphism and AIDs susceptibility for various comparisons

| Stratification | N | | GG vs CC | | | GC vs CC | | GG/GC vs CC | | GG vs GC/CC | | G vs C |  |
| --- | --- | --- | --- | --- | --- | --- | --- | --- | --- | --- | --- | --- | --- |
|  |  | OR (95%CIs) | | *P* | | OR (95%CIs) | *P* | OR (95%CIs) | *P* | OR (95%CIs) | *P* | OR (95%CIs) | *P* |
| Total | 8 | 0.63 (0.51, 0.77) | | <0.001 | 0.78 (0.66, 0.91) | | 0.002 | 0.73 (0.63, 0.84) | <0.001 | 0.73 (0.62, 0.88) | 0.001 | 0.78 (0.71, 0.87) | <0.001 |
| Ethnicity |  |  | |  |  | |  |  |  |  |  |  |  |
| European | 1 | 0.90 (0.59, 1.39) | | 0.646 | 1.06 (0.75, 1.50) | | 0.727 | 1.01 (0.73, 1.41) | 0.932 | 0.87 (0.60, 1.26) | 0.466 | 0.96 (0.78, 1.19) | 0.720 |
| Latin American | 4 | 0.45 (0.33, 0.62) | | <0.001 | 0.64 (0.51, 0.81) | | <0.001 | 0.58 (0.47, 0.72) | <0.001 | 0.58 (0.44, 0.78) | <0.001 | 0.65 (0.56, 0.76) | <0.001 |
| Arab | 2 | 0.71 (0.38, 1.32) | | 0.280 | 0.50 (0.30, 0.82) | | 0.007 | 0.55 (0.34, 0.89) | 0.016 | 1.15 (0.70, 1.91) | 0.578 | 0.84 (0.63, 1.12) | 0.233 |
| Asian | 1 | 0.73 (0.50, 1.08) | | 0.113 | 1.01 (0.74, 1.39) | | 0.928 | 0.92 (0.68, 1.23) | 0.552 | 0.72 (0.51, 1.02) | 0.067 | 0.86 (0.71, 1.05) | 0.143 |
| Disease type |  |  | |  |  | |  |  |  |  |  |  |  |
| T1D | 1 | 0.45 (0.25, 0.81) | | 0.008 | 0.69 (0.44, 1.07) | | 0.097 | 0.61 (0.41, 0.91) | 0.017 | 0.55 (0.32, 0.93) | 0.027 | 0.64 (0.48, 0.86) | 0.003 |
| CD | 2 | 0.76 (0.52, 1.12) | | 0.166 | 0.99 (0.73, 1.34) | | 0.925 | 0.91(0.68, 1.21) | 0.523 | 0.77 (0.55, 1.08) | 0.125 | 0.88 (0.73, 1.07) | 0.197 |
| MS | 1 | 0.74 (0.34, 1.61) | | 0.454 | 0.44 (0.24, 0.84) | | 0.012 | 0.51 (0.28, 0.94) | 0.032 | 1.30 (0.69, 2.43) | 0.415 | 0.85 (0.60, 1.22) | 0.380 |
| RA | 1 | 0.45 (0.27, 0.76) | | 0.003 | 0.57 (0.39, 0.84) | | 0.004 | 0.53 (0.37, 0.76) | 0.001 | 0.61 (0.37, 0.98) | 0.041 | 0.63 (0.49, 0.81) | <0.001 |
| SLE | 2 | 0.67 (0.48, 0.94) | | 0.020 | 0.89 (0.68, 1.17) | | 0.411 | 0.82 (0.64, 1.05) | 0.120 | 0.72 (0.53, 0.96) | 0.028 | 0.82 (0.69, 0.97) | 0.021 |
| MG | 1 | 0.65 (0.23, 1.84) | | 0.421 | 0.78 (0.66, 0.91) | | 0.243 | 0.62 (0.28, 1.38) | 0.243 | 0.93 (0.40, 2.16) | 0.861 | 0.82 (0.51, 1.32) | 0.414 |

N indicates the number of studies involved; OR: odds ratios; *P*: *P*-value of Z-test for OR; T1D: type 1 diabetes; CD: celiac disease; MS: multiple sclerosis; RA: rheumatoid arthritis; SLE: systemic lupus erythematosus; MG: myasthenia gravis
