# Supplementary material for: Association of NLRP3 rs35829419 and rs10754558 Polymorphisms With Risks of Autoimmune Diseases: A Systematic Review and Meta-Analysis
Source: Front Genet. 2021 Jul 22;12:690860. doi: 10.3389/fgene.2021.690860 (PMC8340881; doi:10.3389/fgene.2021.690860)
Supplement: Supplementary file 3 [file Table_3.doc]

Table S3. Quality assessment of the included studies

| Author, year | Selection | Comparability | Exposure | Total score |
| --- | --- | --- | --- | --- |
| Juneblad,2020 | 3 | 2 | 3 | 8 |
| Smigoc, 2019 | 3 | 2 | 3 | 8 |
| Smigoc, 2019 | 3 | 2 | 3 | 8 |
| Pontillo, 2010 | 3 | 2 | 3 | 8 |
| Pontillo, 2010 | 3 | 1 | 3 | 7 |
| Pontillo, 2010 | 3 | 2 | 3 | 8 |
| Pontillo, 2010 | 3 | 2 | 3 | 8 |
| Imani,2018 | 3 | 1 | 3 | 7 |
| Imani,2018 | 3 | 1 | 3 | 7 |
| Addobbati,2018 | 3 | 1 | 3 | 7 |
| Addobbati,2018 | 3 | 2 | 3 | 8 |
| Jenko,2016 | 3 | 1 | 3 | 7 |
| Ben,2012 | 4 | 1 | 3 | 8 |
| Ben,2012 | 4 | 2 | 3 | 9 |
| Kastbom,2010 | 4 | 2 | 3 | 9 |
| Pontillo,2011 | 3 | 2 | 3 | 8 |
| Pontillo,2011 | 3 | 2 | 3 | 8 |
| Su,2020 | 3 | 1 | 3 | 7 |
| Pontillo,2012 | 3 | 1 | 3 | 7 |
| Pontillo,2012 | 3 | 2 | 3 | 8 |
| Agah,2021 | 3 | 2 | 3 | 8 |
| Agah,2021 | 3 | 2 | 3 | 8 |
